# Low-Density-Lipoprotein Cholesterol and Mortality Outcomes Among Healthy Older Adults: A Post Hoc Analysis of ASPREE Trial

**DOI:** 10.1093/gerona/glad268

**Published:** 2023-12-01

**Authors:** Zhen Zhou, Andrew M Tonkin, Andrea J Curtis, Anne Murray, Chao Zhu, Christopher M Reid, Jeff D Williamson, Joanne Ryan, John J McNeil, Lawrence J Beilin, Michael E Ernst, Nigel Stocks, Paul Lacaze, Raj C Shah, Robyn L Woods, Rory Wolfe, Seana Gall, Sophia Zoungas, Suzanne G Orchard, Mark R Nelson

**Affiliations:** School of Public Health and Preventive Medicine, Monash University, Melbourne, Victoria, Australia; Menzies Institute for Medical Research, University of Tasmania, Hobart, Tasmania, Australia; School of Public Health and Preventive Medicine, Monash University, Melbourne, Victoria, Australia; School of Public Health and Preventive Medicine, Monash University, Melbourne, Victoria, Australia; Berman Center for Outcomes and Clinical Research, Hennepin Healthcare Research Institute, Division of Geriatrics, Department of Medicine Hennepin HealthCare, Minneapolis, Minnesota, USA; University of Minnesota, Minneapolis, Minnesota, USA; Department of Neuroscience, Central Clinical School, Monash University, Melbourne, Victoria, Australia; School of Population Health, Curtin University, Perth, Western Australia, Australia; Sticht Center on Aging and Alzheimer’s Prevention, Section on Gerontology and Geriatric Medicine, Department of Internal Medicine, Wake Forest School of Medicine, Winston-Salem, North Carolina, USA; School of Public Health and Preventive Medicine, Monash University, Melbourne, Victoria, Australia; School of Public Health and Preventive Medicine, Monash University, Melbourne, Victoria, Australia; School of Medicine, Royal Perth Hospital, University of Western Australia, Perth, Western Australia, Australia; Department of Pharmacy Practice and Science, College of Pharmacy, The University of Iowa, Iowa City, Iowa, USA; Department of Family Medicine, Carver College of Medicine, The University of Iowa, Iowa City, Iowa, USA; Discipline of General Practice, University of Adelaide, Adelaide, South Australia, Australia; School of Public Health and Preventive Medicine, Monash University, Melbourne, Victoria, Australia; Department of Family Medicine and Rush Alzheimer’s Disease Center, Rush University Medical Center, Chicago, Illinois, USA; School of Public Health and Preventive Medicine, Monash University, Melbourne, Victoria, Australia; School of Public Health and Preventive Medicine, Monash University, Melbourne, Victoria, Australia; Menzies Institute for Medical Research, University of Tasmania, Hobart, Tasmania, Australia; School of Public Health and Preventive Medicine, Monash University, Melbourne, Victoria, Australia; School of Public Health and Preventive Medicine, Monash University, Melbourne, Victoria, Australia; Menzies Institute for Medical Research, University of Tasmania, Hobart, Tasmania, Australia; (Medical Sciences Section)

**Keywords:** Cardiovascular, Geriatric cardiology, Morbidity, Primary care

## Abstract

**Background:**

The prognostic implication of cholesterol levels in older adults remains uncertain. This study aimed to examine the relationship between low-density-lipoprotein cholesterol (LDL-c) and mortality outcomes in older individuals.

**Methods:**

This post hoc analysis examined the associations of LDL-c levels with mortality risks from all-cause, cardiovascular disease (CVD), cancer, and combined non-CVD/noncancer conditions in a cohort of individuals aged ≥65 years from the ASPirin in Reducing Events in the Elderly trial (NCT01038583). At baseline, participants had no diagnosed dementia, physical disability, or CVD, and were not taking lipid-lowering agents. Outcome analyses were performed using multivariable Cox models.

**Results:**

We analyzed 12 334 participants (mean age: 75.2 years). Over a median 7-year follow-up, 1 250 died. Restricted cubic splines found a U-shaped relation for LDL-c and all-cause mortality, cancer mortality, and noncancer/non-CVE mortality (nadir: 3.3–3.4 mmol/L); the risk of CVD mortality was similar at LDL-c below 3.3 mmol/L and increased above 3.3 mmol/L. Similar trends were observed in analyses modeling LDL-c by quartiles. When modeling LDL-c as a continuous variable, the risk of all-cause mortality, cancer mortality, and noncancer/non-CVD mortality was decreased by 9%, 16%, and 18%, respectively, per 1-mmol/L higher LDL-c, and the risk of CVD mortality was increased by 19% per 1-mmol/L higher LDL-c. Reduced all-cause and non-CVD/noncancer mortality risks were only significant in males but not females (*p*_interaction_ < .05).

**Conclusions:**

There were U-shaped relationships between LDL-c and all-cause mortality, cancer mortality, and noncancer/non-CVD mortality in healthy older adults. Higher LDL-c levels were associated with an increased risk of CVD mortality. Future studies are warranted to confirm our results.

The clinical guidelines for lipid management for the primary prevention of cardiovascular disease (CVD) recommend the use of lipid-lowering treatment in older adults aged between 65 and 75 years at high CVD risk based on a plethora of evidence showing the benefits of the treatment of CVD in this age group (1,2). However, no recommendation has been made in terms of lipid-lowering treatment use in people older than 75 years. There is a perception that a low low-density-lipoprotein cholesterol (LDL-c) level is a warning sign of serious diseases and may be linked to high mortality in older adults. This is a major source of confusion for clinicians when comparing the effects of lipids as exposures and the effects of treating lipids in this age group. Most previous studies investigating associations between total and LDL-c and all-cause mortality have found an inverse association, with others showing no association (3–8). Numerous limitations challenge the strength of evidence from these studies. These include unstructured study cohorts with limited ability to control for residual confounding, short follow-up periods, and lack of power due to small sample sizes. In addition, ascertainment of outcomes is reliant on the International Classification of Diseases (ICD) coding that is subject to misclassification bias, and analyses are based on outdated patient data that do not reflect contemporary treatment patterns and the fact that contemporary trends in management of risk factors are better now than previously.

To address these limitations, we investigated longitudinal associations between baseline LDL-c and all-cause and cause-specific mortality outcomes in a large-scale, contemporary, well-characterized, community-based cohort of older individuals who were followed for a median of 7 years (9–12). We chose baseline LDL-c as our study exposure, as it is the target lipid metric in most clinical guidelines for lipid management.

## Method

### Study Design and Participants

This study is exempt from ethics review as only existing nonidentifiable data were used. This analysis included participants in the ASPirin in Reducing Events in the Elderly (ASPREE) study who had LDL-c data and were lipid-lowering agent naive at baseline (9–12). ASPREE was a double-blinded, randomized, placebo-controlled trial of daily low-dose aspirin. From March 2010 through December 2014, 19 114 community-dwelling subjects from Australia (87.4%) and the United States (12.6%) were recruited, who were aged ≥70 years (≥65 for U.S. ethnic minorities), with no prior history of CVD, dementia, or major physical disability at trial entry. The intervention phase of the trial ended in June 2017. Over 80% of study participants agreed to be followed for a further 5-year post-trial (ASPREE-XT). For this analysis, the end of the follow-up was the second ASPREE-XT annual visit (the last visit was completed in August 2019). The study design and principal findings of the ASPREE trial are published elsewhere (9–12). The description of ASPREE-XT is available on the ASPREE website (https://aspree.org/).

### Exposure and Outcomes

In ASPREE, at baseline, participants fasting high-density-lipoprotein cholesterol (HDL-c), total cholesterol, and triglycerides were measured in a clinic or local pathology center. LDL-c was calculated using the Friedewald equation except where triglycerides were too high, and in the latter case, direct LDL-c measurements were made.

Outcome measures were all-cause mortality, CVD mortality, cancer mortality, and combined non-CVD/noncancer mortality. Non-CVD/noncancer mortality was analyzed in total and by subcategory. CVD mortality was defined as coronary heart disease death, stroke death, and deaths due to any cardiovascular causes. Death was identified during the course of trial regular activity, or notified by the participant’s next of kin or a close contact during the follow-up. Notification of all death cases required confirmation from at least 2 independent sources, like family members, primary care physician, or public death notice. The cause of death was adjudicated by a panel with relevant clinical expertise blinded to the ASPREE randomized allocation (9).

### Covariates

Covariates were selected a priori that included age, sex, race/country, body mass index, HDL-c, triglycerides (log transformed), smoking status, alcohol consumption, years of education, blood pressure, and use of antihypertensive medication, diabetes, frailty (11), and randomized treatment assignment (aspirin/placebo). Data on confounders were more than 99.6% complete. Missing data were filled with age and sex-adjusted mean values.

### Statistical Analysis

Cox proportional hazards regression models adjusting for all covariates listed in Table 1 were used for the outcome analyses of LDL-c. LDL-c was modeled as either a categorical (by quartile) or continuous variable. When modeling LDL-c as a categorical variable, for each individual analysis, the quartile group associated with a lowest death risk was selected as the reference. The proportional hazards assumption was checked by Schoenfeld residuals. The outcome analyses were repeated separately for men and women, and for age <75 years and age ≥75 years. The *p* for interaction was obtained from a multiplicative term of LDL-c with sex or age. Restricted cubic splines with 3 prespecified knots based on Harrell’s recommended percentiles (10th, 50th, and 90th) were plotted to visualize the potentially nonlinear relationship between LDL-c and study outcomes. Thin-plate smoothing splines were plotted to explore the potential change in the association between LDL-c (treated as a binary variable) and outcomes with age.

**Table 1.** Baseline Characteristics of Participants in by Sex

Characteristic	Males (*n* = 5 681)	Females (*n* = 6 653)
Age	75.1 ± 4.6	75.2 ± 4.7
Race/country		
White Australian	5 062 (89%)	5 612 (84%)
White American	216 (4%)	462 (7%)
Non-White (Australia/U.S. combined)	403 (7%)	578 (9%)
Education		
<12 years	2 469 (43%)	2 916 (44%)
≥12 years	3 212 (57%)	3 737 (56%)
LDL cholesterol (mmol/L)	3.2 ± 0.8	3.4 ± 0.8
HDL cholesterol (mmol/L)	1.4 ± 0.4	1.8 ± 0.5
Triglycerides (mmol/L)	1.3 ± 0.6	1.3 ± 0.6
Smoking status		
Nonsmoker	2 552 (45%)	4 346 (65%)
Ex-smoker	2 869 (51%)	2 092 (31%)
Current smoker	260 (5%)	215 (3%)
Drinking status		
Never	571 (10%)	1 495 (22%)
Ex-drinker	399 (7%)	306 (5%)
Current drinker	4 711 (83%)	4 852 (73%)
Blood pressure		
<140/90 and not on med	1 596 (28%)	2 045 (31%)
SBP/DBP < 140/90 mmHg, on med	980 (17%)	1 572 (24%)
SBP/DBP ≥ 140/90 mmHg, no med	1 726 (30%)	1 433 (22%)
SBP/DBP ≥ 140/90 mmHg, on med	1 379 (24%)	1 603 (24%)
Diabetes		
No diabetes	5 280 (93%)	6 304 (95%)
Yes, on med	176 (3%)	145 (2%)
Yes, not on med	225 (4%)	204 (3%)
Chronic kidney disease		
No	4 049 (71%)	4 691 (71%)
Yes	1 262 (22%)	1 531 (23%)
Uncertain	370 (7%)	431 (6%)
BMI categories		
Under/normal weight (<25 kg/m^2^)	1 408 (25%)	2 212 (33%)
Overweight (25–29.9 kg/m^2^)	2 951 (52%)	2 517 (38%)
Obese (≥30 kg/m^2^)	1 322 (23%)	1 924 (29%)
Frailty		
No	3 501 (62%)	3 860 (58%)
Prefrail	2 092 (37%)	2 633 (40%)
Frail	88 (2%)	160 (2%)
Randomized to aspirin	2 822 (50%)	3 302 (50%)

*Notes*: Data are *n* (%) or mean ± *SD*. Non-White was comprised of Black, Hispanic/Latino, other race/ethnicities including Australian aborigine/Torres Strait islander, native American, more than 1 race, native Hawaiian/Pacific Islander, and those who were not Hispanic and who did not state their ethnicity/race. Hypertension was defined as “on treatment” for high BP or BP > 140/90 mmHg at study entry; diabetes mellitus was defined from self-report or fasting glucose ≥126 mg/dL or on treatment for diabetes. Chronic kidney disease was defined as estimated glomerular filtration rate <60 mL/min/1.73 m^2^ or urinary albumin to creatinine ratio ≥30 mg/mmol; “Prefrail” included anyone with 1 or 2 criteria and “Frail” included anyone with 3 or more criteria of the adapted Fried frailty criteria. These included body weight (BMI < 20 kg/m^2^), strength (hand grip in lowest 20% of participants by sex and Fried-defined sex-specific BMI categories), exhaustion (taken from the self-reported CES-D-10 responses) walking speed (3 m gait speed in lowest 20% of participants by sex and Fried-defined sex-specific height categories) and physical activity (taken from the Self-Reported Life Questionnaire) (11). BMI = body mass index; BP = blood pressure; CES-D = Center for Epidemiologic Studies Short Depression Scale; DBP = diastolic blood pressure; HDL = high-density-lipoprotein; LDL = low-density-lipoprotein; SBP = systolic blood pressure.

We conducted several sensitivity analyses. To address confounding by indication, we repeated the outcome analyses by excluding participants who died in the 5 years after baseline, as suggested in previous literature (13,14). The presence of reverse causality was also tested by conducting an analysis for the association between LDL-c and non-CVD mortality outcomes by baseline frailty status, in a subcohort of participants with LDL-c concentration in the lower 50th percentile (<3.3 mmol/L [127.6 mg/dL]). We pooled all death events due to non-CVD causes in this analysis due to the small event number for each individual non-CVD mortality outcome in the frailty group. We also repeated the main analyses for the 3 cause-specific mortality outcomes using a Fine–Gray semiparametric proportional subdistribution hazards model to consider competing events. To address the impact of change in LDL-c levels over time, we repeated the outcome analyses with the baseline LDL-c replaced by the mean of LDL-c concentrations measured at baseline, the first and second in-trial annual follow-up visits.

Analyses were performed using Stata/SE version 16.0 (StataCorp LLC, College Station, TX), and R (R Foundation for Statistical Computing, Vienna, Austria), version 1.1.456, with “*mgcv*” package to produce thin-plate smoothing splines.

## Results

### Baseline Characteristics

We included 12 334 participants who were not taking baseline lipid-lowering medication [mean (standard deviation {*SD*} age: 75.2(4.6) years; 54% females]. Compared with male participants, female participants were less likely to be White Australians, ex- and current smokers, and drinkers, and had a lower prevalence of hypertension and diabetes, but had higher LDL-c and HDL-c and prevalence of obesity. (Table 1) Histograms of the LDL-c concentration in the entire cohort and by sex are shown in Supplementary Figure 1.

### Associations Between LDL-c and Mortality

**Figure 1.**
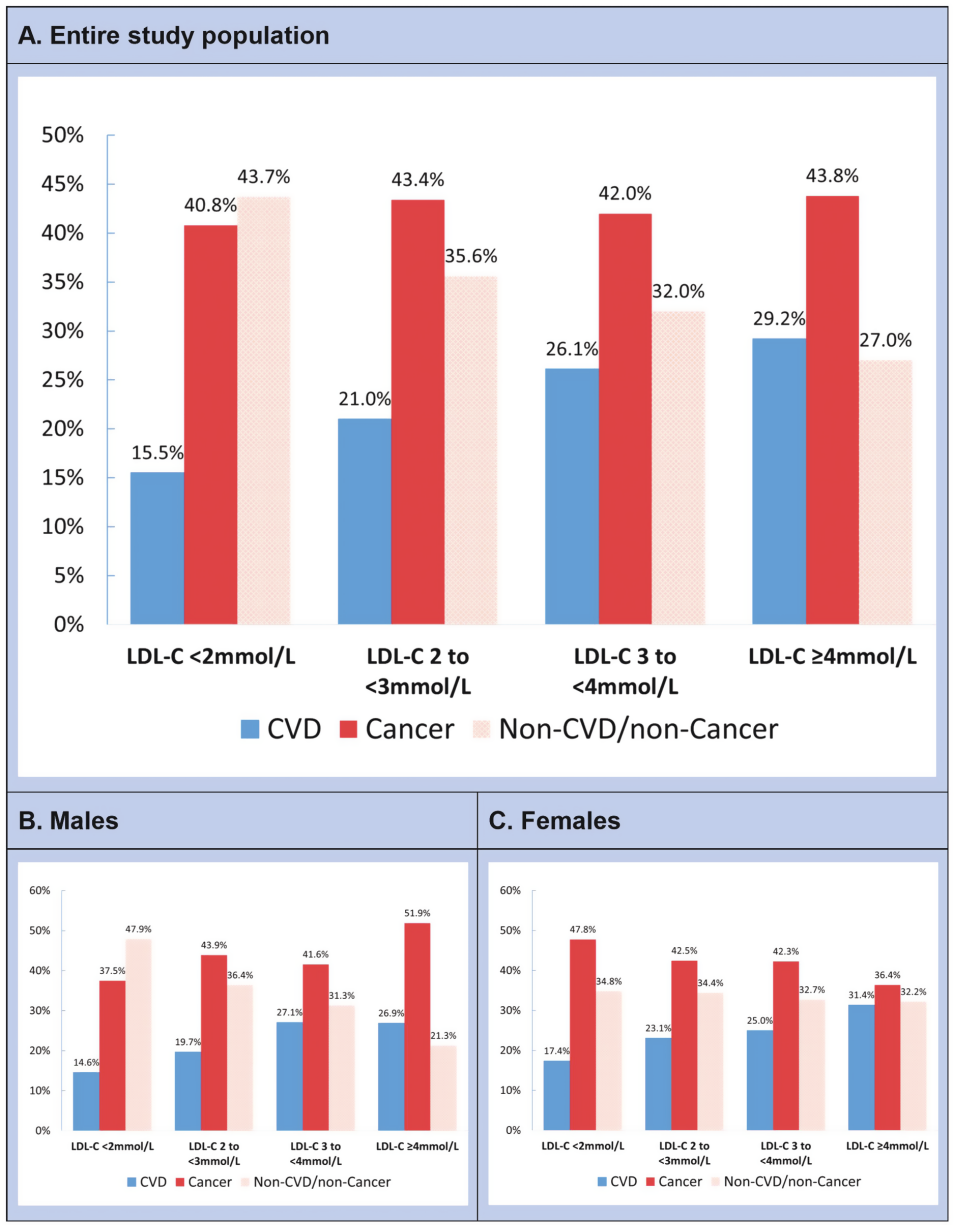
Distribution of deaths across LDL-c in the entire study population and by sex. The number of deaths in the LDL-c of <2 mmol/L (*n* = 451), 2–<3 mmol/L (*n* = 3 542), 3–<4 mmol/L (*n* = 5 957), and ≥4 mmol/L (*n* = 2 384) categories were 71, 424, 529, and 226, respectively. LDL-c = low-density-lipoprotein cholesterol.

A total of 1 250 (10.1%) participants died over a median (interquartile range [IQR] follow-up of 6.9 (5.7–8.0) years, with 304 (24.3%) dying from CVD, 534 (42.7%) from cancer, and 412 (33.0%) from non-CVD/noncancer conditions. For CVD mortality, 133 died due to coronary heart disease, 19 due to hemorrhagic stroke, 73 due to nonhemorrhagic strokes, and 79 due to other CVD causes. Figure 1 shows the distribution of each study outcome across LDL-c categories. There was a general higher proportion of CVD death and a lower proportion of non-CVD/noncancer death with increasing LDL-c in both males and females. Cancer caused most death events at all LDL-c levels in males and females, except for the lowest level, ranging from 40.8% to 43.8%.

**Figure 2.**
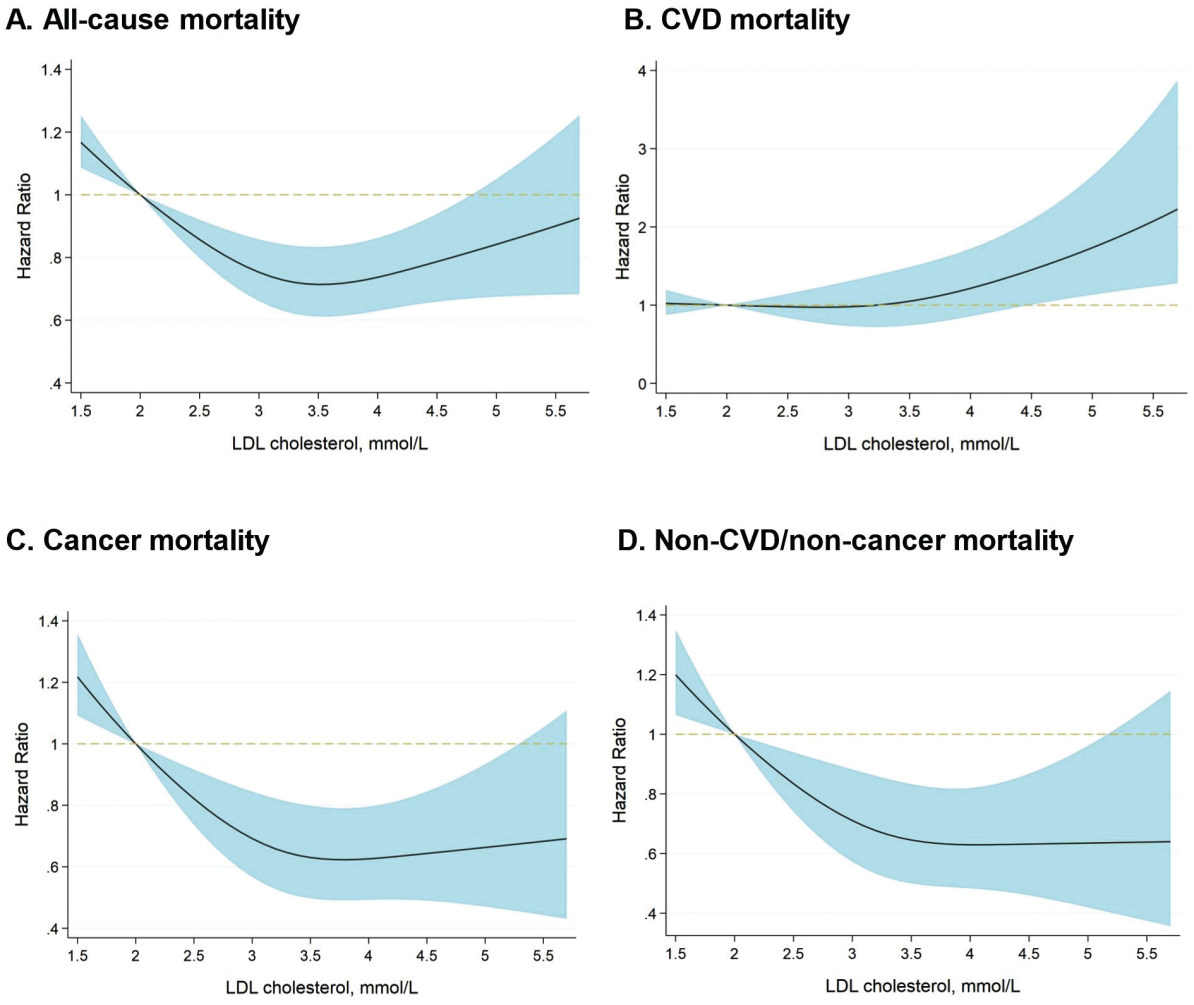
Restricted cubic spline for the fully adjusted HR of each mortality outcome for LDL-c. The reference was LDL-c of 2 mmol/L (77.3 mg/dL). Adjustment was made on all variables listed in Table 1. Abbreviations as in Table 2 and Figure 1.

We observed a U-sharped relationship for LDL-c and all-cause mortality, where the nadir was around LDL-c 3.3 mmol/L (Figure 2). The hazard ratios (HRs) for cancer mortality and non-CVD/noncancer mortality were similar at LDL-c levels above 3.4 mmol/L and increased steeply below the LDL-c level of 3.4 mmol/L. The HRs for CVD mortality were similar at LDL-c below 3.3 mmol/L and increased above the LDL-c level of 3.3 mmol/L. In analyses grouping participants by LDL-c quartiles, similar trends were observed in the entire study population and by sex (Table 2). In the overall study cohort, the third quartile of LDL-c (3.4–3.8 mmol/L) was associated with the lowest risk of all-cause mortality, cancer mortality, and combined noncancer/non-CVD mortality. The risk of CVD mortality was found to be lowest in the first quartile group of LDL-c (0.5–2.8mmol/L).

**Table 2.** Hazard Ratios for Mortality Outcomes by the Quartiles of LDL Cholesterol

LDL Cholesterol Categories by Quartiles	All Cause	CVD	Cancer	Non-CVD/Noncancer
	Fully Adjusted HR (95% CI)			
Total				
1st quartile (0.5–2.8 mmol/L)	1.33 (1.13–1.56)	1 (Ref)	1.56 (1.22–2.01)	1.51 (1.13–2.00)
2nd quartile (2.9–3.3 mmol/L)	1.17 (0.99–1.39)	1.03 (0.75–1.41)	1.29 (0.99–1.68)	1.29 (0.96–1.75)
3rd quartile (3.4–3.8 mmol/L)	1 (Ref)	1.16 (0.84–1.60)	1 (Ref)	1 (Ref)
4th quartile (3.9–9.9 mmol/L)	1.16 (0.97–1.38)	1.32 (0.95–1.82)	1.19 (0.91–1.57)	1.13 (0.81–1.56)
Males				
1st quartile (0.5–2.7 mmol/L)	1.58 (1.27–1.98)	1.04 (0.68–1.60)	1.66 (1.18–2.32)	2.18 (1.44–3.31)
2nd quartile (2.8–3.2 mmol/L)	1.36 (1.08–1.71)	1 (Ref)	1.42 (1.00–2.01)	1.78 (1.16–2.74)
3rd quartile (3.3–3.7 mmol/L)	1 (Ref)	1.08 (0.69–1.69)	1 (Ref)	1 (Ref)
4th quartile (3.8–9.9 mmol/L)	1.23 (0.97–1.57)	1.22 (0.78–1.90)	1.30 (0.91–1.87)	1.22 (0.76–1.96)
Females				
1st quartile (0.6–2.8 mmol/L)	1.08 (0.86–1.36)	1 (Ref)	1 (Ref)	1.15 (0.78–1.71)
2nd quartile (2.9–3.4 mmol/L)	1 (Ref)	1.20 (0.74–1.93)	0.84 (0.60–1.18)	1 (Ref)
3rd quartile (3.5–3.9 mmol/L)	1.02 (0.80–1.29)	1.29 (0.79–2.13)	0.77 (0.53–1.11)	1.11 (0.73–1.70)
4th quartile (4.0–7.0 mmol/L)	1.03 (0.81–1.32)	1.60 (0.98–2.64)	0.67 (0.45–1.00)	1.15 (0.74–1.79)

*Notes*: Adjustment was made on all variables listed in Table 1. To convert LDL cholesterol in mmol/L to mg/dL, multiply by 38.67. CI = confidence interval; CVD = cardiovascular disease; HR = hazard ratio; LDL = low-density lipoprotein.

Table 3 shows the analysis results of adjusted HRs of each mortality outcome for LDL-c on a continuous scale, in which the resulting linear effects of LDL-c on hazards ignored curvature but indicated a generally reduced mortality risk (increased risk for CVD mortality) with higher LDL-c. Each 1-mmol/L higher LDL-c was associated with a lower risk of all-cause mortality (HR = 0.91, 95% confidence interval [CI] 0.84–0.98, *p* = .01), cancer mortality (HR = 0.84, 95% CI 0.74–0.94, *p* = .002), and non-CVD/noncancer mortality (HR = 0.82, 95% CI 0.72–0.93, p = .003) but a higher risk of CVD mortality (HR = 1.19, 95% CI 1.03–1.39, *p* = .02). The analysis of non-CVD/noncancer mortality subcategories found that lower LDL-c was particularly associated with higher death rates due to chronic obstructive pulmonary disease (COPD), sepsis/infection, and liver disease (Supplementary Table 1). In addition, for all-cause mortality, the adjusted HR (95% CI) per 1 mmol/L increase in LDL-c was 0.91 (0.84–0.98) for Australian participants and 0.95 (0.75–1.20) for the U.S. participants. There is no modifying effect of country on the association between LDL-c and all-cause mortality (*p* for interaction = .86).

**Table 3.** Hazard Ratios of Mortality Outcomes Per 1 mmol/L Increase in LDL Cholesterol

	Complete Follow-Up (*N* = 12 334)			Deaths in the First 5 Years Excluded (*N* = 11 627)		
Cause of Death	Events (rate^*^)	Fully Adjusted HR (95% CI) Per 1 mmol/L Higher LDL Cholesterol	*p* Value	Events	Fully Adjusted HR (95% CI) Per 1 mmol/L Higher LDL Cholesterol	*p* Value
All cause	1 250 (15.2)	0.91 (0.84–0.98)	.01	545	1.01 (0.90–1.13)	.85
CVD	304 (3.7)	1.19 (1.03–1.39)	.02	128	1.18 (0.93–1.49)	.17
Cancer	534 (6.5)	0.84 (0.74–0.94)	.002	205	1.04 (0.86–1.25)	.70
Non-CVD/noncancer	412 (5.0)	0.82 (0.72–0.93)	.003	212	0.90 (0.75–1.08)	.26

^*^Rate was incidence rate per 1 000 person-years. Adjustment was made on all variables listed in Table 1. Abbreviations as in Table 2.

### LDL-c and Mortality Outcomes by Sex

Sex modified the association between LDL-c and all-cause mortality (*p* for interaction = .04) and non-CVD/noncancer mortality (*p* for interaction = .006) At lower LDL-c levels, males had an increased risk of all-cause mortality and non-CVD/noncancer mortality but not in females. (Table 2 and Supplementary Table 2).

### LDL-c and Mortality Outcomes by Age

Age modified the association between LDL-c and CVD mortality (*p* for interaction = .04; Supplementary Table 2). Participants aged ≤75 years with higher LDL-c were at a greater CVD mortality risk than those aged >75 years. Consistent with this result, the splines revealed an attenuated relationship between LDL-c and CVD mortality with older age, especially at 80 years or older (Supplementary Figure 2).

### Sensitivity Analysis

In a sensitivity analysis excluding deaths that were occurred within the initial 5 years of follow-up, the HRs (95%CI) for all-cause, CVD, cancer, and non-CVD/noncancer mortality were 1.01 (0.90–1.13), 1.18 (0.93–1.49), 1.04 (0.86–1.25), and 0.90 (0.75–1.08), respectively (Table 3). Limiting the analysis to participants whose LDL-c below the 50th percentile of the study population, the HR (95% CI) per 1 mmol/L increase for pooled non-CVD mortality outcomes in nonfrail, prefrail, and frail participants was 0.87 (0.66–1.14), 0.80 (0.64–1.02), and 0.20 (0.09–0.42), respectively (global *p* value for interaction = .06). Results yielded from the Fine and Gray competing risk models for the 3 cause-specific mortality outcomes were essentially the same as those from the main analysis (data not shown). In other sensitivity analyses replacing baseline LDL-c values with the mean LDL-c measurement at the baseline, first, and second annual visits, results were comparable to those from the main analysis, with HRs (95% CI) for all-cause, CVD, cancer, non-CVD/noncancer mortality of 0.90 (0.83–0.98), 1.24 (1.06–1.45), 0.82 (0.73–0.93), and 0.80 (0.69–0.92), respectively.

## Discussion

In this post hoc analysis of a randomized trial including 12 334 older adults who were free of CVD and did not take any lipid-lowering agent at baseline, we found curvilinear relationships between LDL-c level, all-cause mortality, cancer mortality, and non-CVD/noncancer mortality, with the greatest risk of death at the lower end of the LDL-c values. Higher LDL-c was associated with a generally greater CVD mortality risk in our study population.

U-shaped relationships between LDL-c and all-cause mortality in older populations have been reported in many previous studies (15,16), as has an inverse association for non-CVD mortality (17,18). The previous studies concluded that the observed associations were driven by “confounding by indication,” implying that the relationship between cholesterol and mortality outcomes is biased by the unmeasured or unobserved pre-existing illnesses and conditions that act to influence lipid metabolism (19). Most of these studies, however, lacked data to support their assumptions that the exposure to conditions that lower LDL-c levels was driving the association rather than the LDL-c per se. In our study, we investigated the possibility of confounding by indication in a sensitivity analysis by excluding deaths that occurred in the first 5 years of follow-up. We assumed that, if occult disease was present at baseline, it would lead to death during this timeframe. The results showed no significant difference between LDL-c and all-cause, cancer, and non-CVD/noncancer mortality and CVD mortality. This indicates that reverse causality might contribute to the observing associations for mortality outcomes in the main analyses. That being said, our data can neither confirm nor deny a real U-shaped relationship between LDL-c and non-CVD mortality outcomes. Future studies with a better approach to eliminating possible confounding are warranted to help disentangle the complex relationships between LDL-c and different mortality outcomes. Such “confounding” can also be present in real-life clinical practice. Low cholesterol levels in older individuals could be indicative of underlying issues like wasting diseases, frailty, or malnutrition, all of which are associated with an increased mortality risk. It’s crucial to distinguish between healthy low cholesterol levels, which imply a reduced CVD risk, and low-level stemming from debilitating diseases.

We further found that low LDL-c was associated with a higher risk of death for sepsis/infection, COPD, and liver disease, which to some degree supports our assumption regarding the confounding by indication. Low cholesterol may indicate the presence of comorbidities and frailty that are thought to increase the risk of infection (20,21). Similarly, wasting diseases such COPD, cancers, and liver disease, which could reduce the production and secretion of lipoproteins (13), are common in older people and often linked to deteriorating nutritional status and accelerated terminal cholesterol decline in those who approach death (6,22,23). In our sensitivity analysis limited to participants whose LDL-c is below the 50th percentile of the study population, there was modest evidence supporting that the LDL-c and non-CVD mortality paradox only exists in frail older individuals but not in those without frailty. Further, the development and progression of COPD, liver disease, and new-onset cancers are often triggered by smoking and alcohol abuse. In our study, male participants were almost twice as likely as female participants to be former and current smokers and more likely to be current drinkers. This difference might partially explain why the inverse association between low LDL-c and high all-cause and non-CVD/noncancer mortality in our study was maintained in males but not in females. Another explanation is that the proportion of non-CVD mortality at low LDL-c levels (<3 mmol/L) is lower in females than males. Similar findings of sex differences in the relationship between cholesterol and all-cause mortality have also been reported by others (7,24). A report from the National Heart, Lung, and Blood Institute conference also suggested that the relationship between LDL-c and mortality outcomes differed between males and females and was U-shaped in males but flat in females (13).

Consistent with our findings, a large population-based study following 350 977 men aged 35–57 years for 12 years also found that serum cholesterol was inversely associated with death from cancers of the lung, lymphatic, and hematopoietic systems, COPD, and digestive disease (particularly liver disease) (20). Furthermore, a cohort study of 724 adults aged over 85 years who were followed for 10 years reported a lower mortality risk from cancer, infection, and respiratory diseases among individuals with higher total cholesterol levels, and this largely explained their lower risk for overall mortality (6). ASPREE excluded participants who had a life expectancy of 5 years or less, the putative duration of the study. This suggests that subclinical and insidious diseases might lead to reverse causality and are hard to identify in observational studies. Indeed, the CVD death rate in our cohort was lower than the population norm, whereas cancer and non-CVD/noncancer mortality dominated the overall mortality risk and thus was likely the main driver of the observed paradox between low LDL-c and high all-cause mortality.

The evidence for LDL-c and CVD mortality might be less likely influenced by indication bias in this study, due to the adequate adjustment for major CVD risk factors and the analysis of the primary prevention population. However, the survival bias may be at play. Most individuals in whom LDL-c levels could be drivers of CVD mortality may have had their events already at a younger age and would not be part of the study. In contrast, lower cholesterol will lead to lower CVD event rates in middle age and survival into older age where non-CVD diseases then become prominent. This may partially explain the increment in non-CVD mortality and attenuation of CVD mortality with LDL-c levels observed in our study cohort. Interestingly, we found LDL-c of around 3.3 mmol/L to be a value below which the CVD mortality risk was not elevated. This value is well above the commonly recommended level (1.8 mmol/L [70 mg/dL]) for primary prevention of CVD. However, the risk of CVD is more sensitive to LDL-c change than the risk of CVD mortality. The weaker association between higher LDL-c and CVD mortality over the age of 75 agrees with reports by other studies (25,26). This might be explained by the decreased attributable CVD mortality risk in relation to LDL-c, when age is such a dominant risk factor at age >75. A recent analysis using data from the Copenhagen General Population Study in 13 779 individuals aged 70–100 years found that the risks of atherosclerotic CVD and myocardial infarction were increased by 12%–16% and 25%–28% per 1 mmol/L increase in LDL-c during an 8-year follow-up (27). These results are consistent with our data for CVD mortality.

The strengths of our study include the well-characterized, contemporary, community-dwelling cohort of apparently healthy older people, its large sample size, the comprehensive demographic, and clinical information collected on an ongoing basis from participants, rigorous outcome ascertainment and adjudication, and extended follow-up. The sex distribution is close to the population norm which allows for the representativeness of females in the study cohort.

This study has several limitations. First, this was an observational study and thus the causal relationship between LDL-c and study outcomes cannot be established. Second, we did not exclude participants who initiated lipid-lowering treatment during follow-up, as the number of new users was small (22%, *n* = 2 689) and most of them used drugs for secondary prevention purposes (28). Third, the ASPREE participants were apparently healthy compared with the general population of the same age range, therefore, to what degree the findings can be applied to the general population is unknown. Similarly, most of the participants in this study (86.5%, *n* = 10 674) were White Australians, affecting the generalizability of the findings to other racial/ethnic groups and countries. In ASPREE, the ratio of non-CVD death to CVD death was much higher than the population norm, indicating a survivor bias introduced by recruitment criteria, and this could have affected the magnitude and direction of the association between LDL-c and all-cause mortality. Last, this study focused on natural cholesterol levels only, and our study findings cannot be generalized to the populations on lipid-lowering medications. Indeed, the prognostic value of cholesterol has been seen that differs in treated and untreated individuals (18,29).

In conclusion, there was a U-shaped relationship between untreated LDL-c level and all-cause mortality, cancer mortality, and combined noncancer/non-CVD mortality. Higher LDL-c levels were associated with an increased risk of CVD mortality and this association was attenuated with older age. Whether the results for non-CVD mortality outcomes reflect a true relationship or are subject to confounding bias cannot be determined by current data. Prospective studies with better study design, data, and analytic approach to eliminating potential confounding bias are needed to provide more robust evidence.

## Supplementary Material

glad268_suppl_Supplementary_Appendix

## Data Availability

Requests for data access will be via the ASPREE Principal Investigators with details for applications provided through https://aspree.org/aus/for-researchers/ or https://aspree.org/usa/for-researchers/.
