# Supplementary material for: Low-Density-Lipoprotein Cholesterol and Mortality Outcomes Among Healthy Older Adults: A Post Hoc Analysis of ASPREE Trial
Source: J Gerontol A Biol Sci Med Sci. 2023 Dec 1;79(4):glad268. doi: 10.1093/gerona/glad268 (PMC10960624; doi:10.1093/gerona/glad268)
Supplement: glad268_suppl_Supplementary_Appendix [file glad268_suppl_supplementary_appendix.docx]

**Appendix**

**Supplement to:** Low-density-lipoprotein cholesterol and mortality outcomes among healthy older adults: A post-hoc analysis of ASPREE.

**Online Table 1. HRs of sub-categories of non-CVD/non-cancer mortality for per 1 mmol/L increase in LDL cholesterol.**

| **Subcategories of non-CVD/non-cancer death** | **Events (Incidence rate per 1000 person-years)** | **Fully adjusted HR**  **(95% CI)** | **P value** |
| --- | --- | --- | --- |
| COPD | 56 (0.7) | 0.48 (0.33-0.69) | <0.001 |
| Other respiratory diseases^*^ (non-COPD) | 69 (0.7) | 0.96 (0.70-1.33) | 0.82 |
| Sepsis/infection | 37 (0.4) | 0.56 (0.36-0.86) | 0.008 |
| Traumatic | 38 (0.5) | 1.20 (0.77-1.86) | 0.42 |
| Gastrointenstinal disease | 11 (0.1) | 0.94 (0.40-2.22) | 0.88 |
| Liver disease | 9 (0.1) | 0.24 (0.08-0.66) | 0.006 |
| Other causes ^†^ | 192 (2.3) | 1.02 (0.76-1.12) | 0.41 |

*Other respiratory diseases included aspiration pneumonia (n=9), infective pneumonia (n=42), pneumonia – unspecified, other respiratory diseases (n=11). ^†^ Other causes included blood/coagulation disorders (n=1), dementia (n=55), epilepsy (n=1), motor neurone disease (n=7), multi-organ failure (n=7), Parkinson’s disease (n=5), kidney disease (n=13), suicide (n=2), supra nuclear palsy (n=2), non-specific/other/unknown (n=43), other -neurological disorder (n=16), major bleeding (n=24), unascertained- natural causes (n=7), cause of death not adjudicated (n=9). Adjustment was made on all variables listed in Table 1. Abbreviation: COPD, chronic obstructive pulmonary disease; other abbreviations as in Table 2.

**Online Table 2. The associations of LDL cholesterol level on a continuous scale with mortality outcomes, by sex and age.**

| **Cause of death** | **Males (n=5,681, mean age: 75.1 years)** | | | **Females (n=6,653, mean age: 75.2 years)** | | | P interaction |
| --- | --- | --- | --- | --- | --- | --- | --- |
|  | Events (Incidence rate per 1000 person-years) | Fully adjusted HR (95% CI) | | Events (Incidence rate per 1000 person-years) | | Fully adjusted HR (95% CI) |  |
| All-cause | 701 (18.7) | 0.86 (0.77-0.95) | | 549 (12.2) | | 0.98 (0.88-1.10) | 0.048 |
| CVD | 164 (4.4) | 1.14 (0.93-1.41) | | 140 (3.1) | | 1.29 (1.04-1.60) | 0.56 |
| Cancer | 307 (8.2) | 0.85 (0.73-0.99) | | 227 (5.1) | | 0.83 (0.70-0.99) | 0.99 |
| Non-CVD/ non-cancer | 230 (6.1) | 0.71 (0.59-0.85) | | 182 (4.1) | | 0.98 (0.81-1.18) | 0.006 |
| **Cause of death** | **Age<75 years (n=7,199)** | | | **Age ≥75 years (n=5,135)** | | | P interaction |
|  | Events (Incidence rate per 1000 person-years) | | Fully adjusted HR (95% CI) | Events (Incidence rate per 1000 person-years) | Fully adjusted HR (95% CI) | |  |
| All-cause | 423 (8.8) | | 0.91 (0.80-1.04) | 827 (24.2) | 0.91 (0.83-1.00) | | 0.82 |
| CVD | 84 (1.7) | | 1.48 (1.13-1.96) | 220 (6.4) | 1.09 (0.91-1.30) | | 0.04 |
| Cancer | 212 (4.4) | | 0.82 (0.68-0.99) | 322 (9.4) | 0.85 (0.74-0.99) | | 0.63 |
| Non-CVD/non-cancer | 127 (2.6) | | 0.77 (0.60-0.97) | 285 (8.4) | 0.84 (0.72-0.99) | | 0.39 |

Adjustment was made on all variables listed in Table 1 except for stratification variable. Abbreviations as in Table 2.

**Online Figure 1. Baseline LDL cholesterol concentration of the entire study cohort and by sex**

**A. In total**


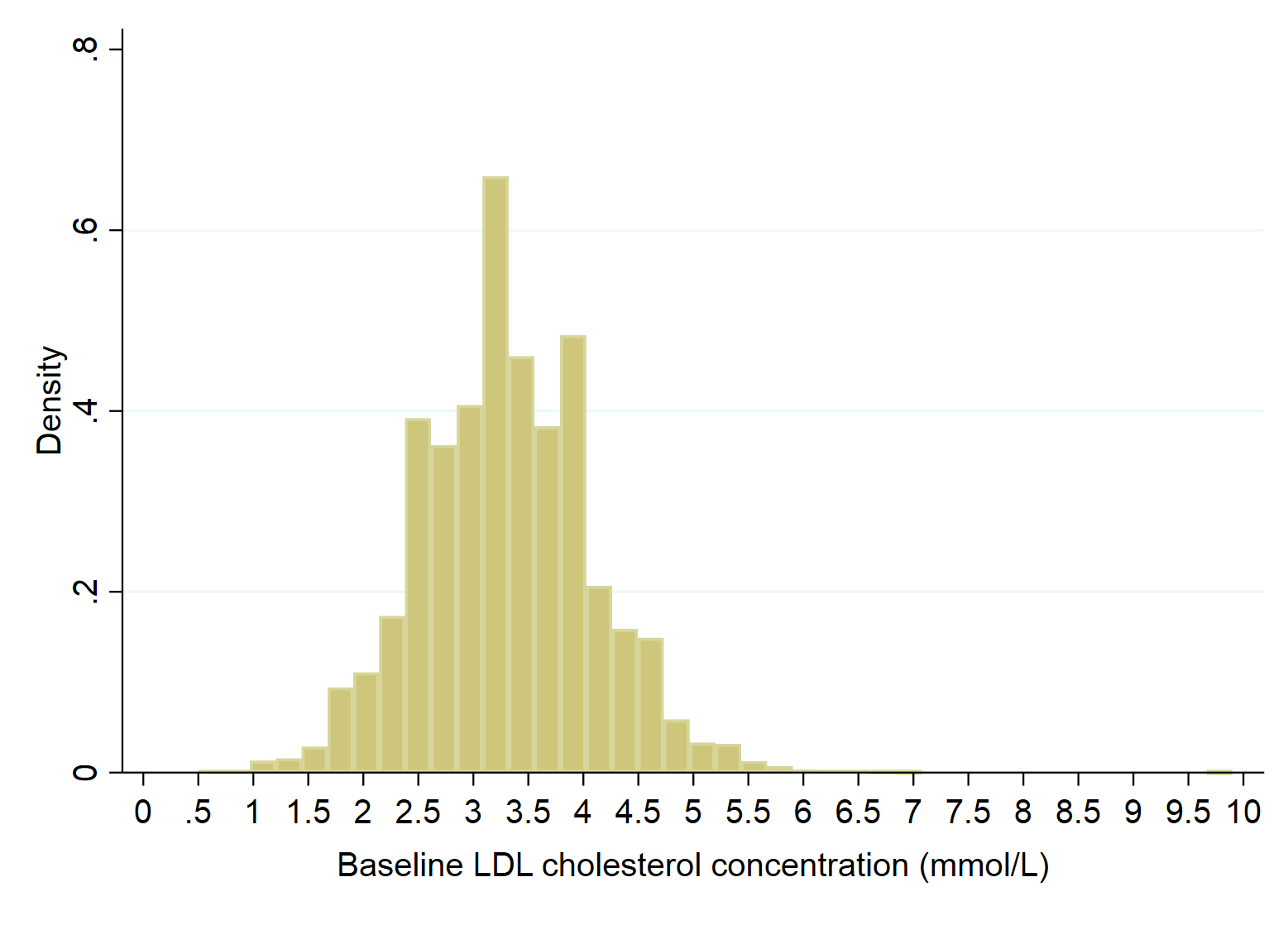


**B. by sex**


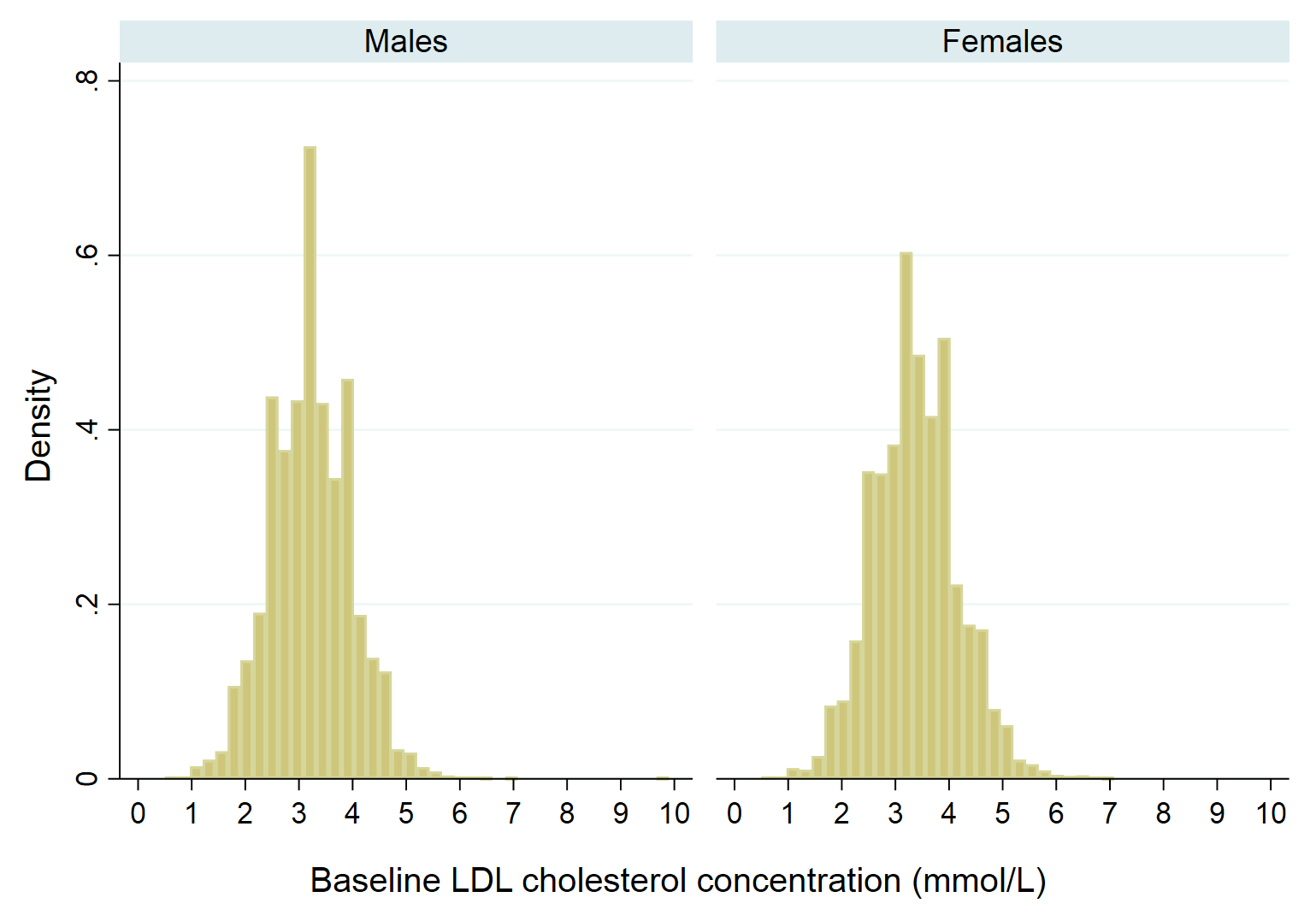


**Online Figure 2.** Relationship between LDL-C and mortality outcomes with increasing age.


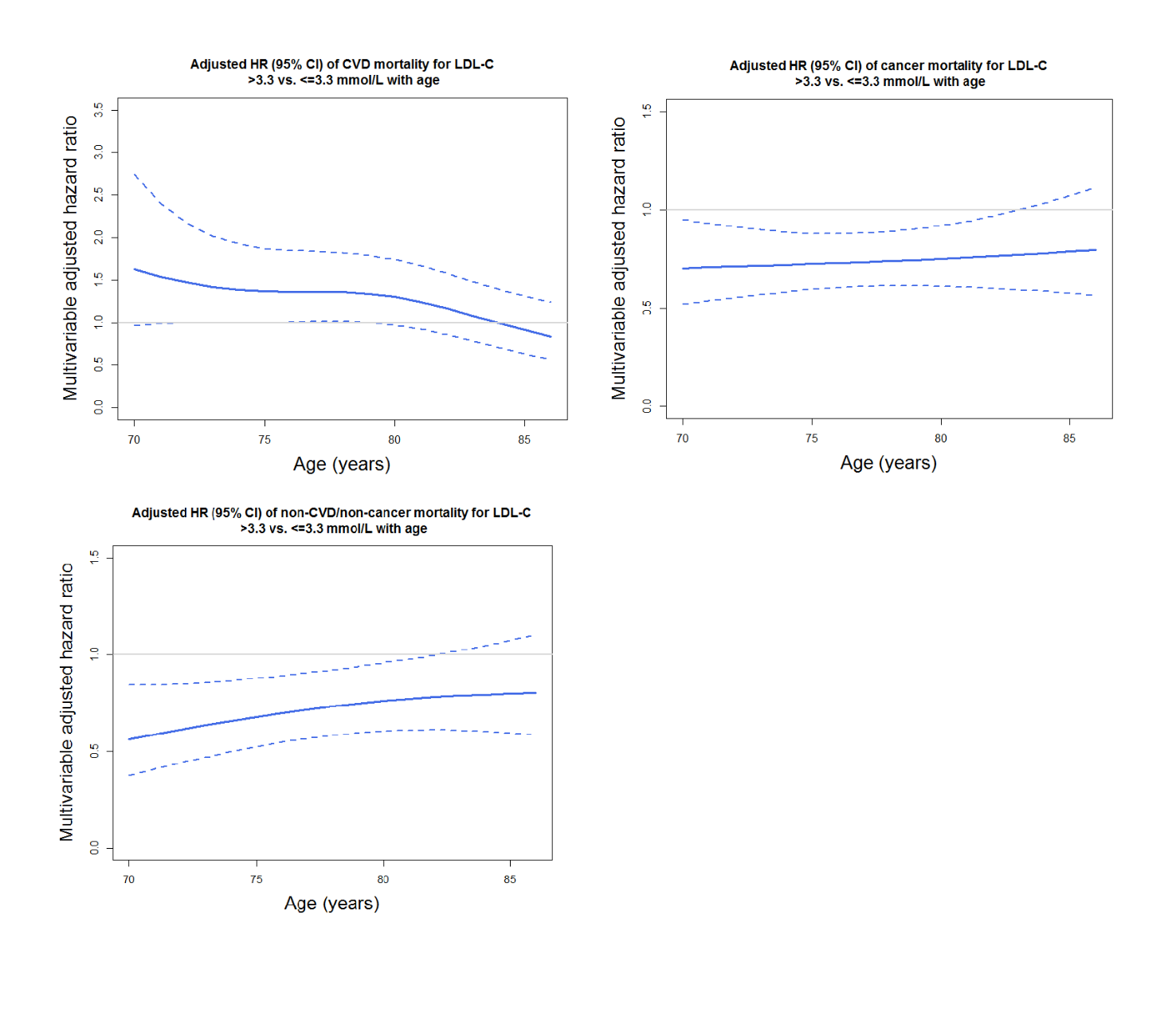


The figure shows thin plate regression spline for HRs of mortality outcomes for LDL-C >3.3 mmol/L (127.6 mg/dL) versus ≤3.3 mmol/L with increasing age at enrolment on a continuous scale. LDL-C of 3.3 mmol/L was used for grouping as it is the median value of LDL-C of the study cohort and the nadir value of the U-shaped relation between LDL-C and all-cause mortality. The hazard ratio was fully adjusted HR of each mortality outcome. Adjustment was made on all variables listed in Table 1. Abbreviations as in Table 2 and Figure 1.
